# Free Volume Space of Polymers as a New Functional Nanospace: Synthesis of Guest Polymers

**DOI:** 10.1002/marc.202400980

**Published:** 2025-01-30

**Authors:** Sayaka Hirai, Tomoki Sakuma, Yuki Tokura, Hiroaki Imai, Ryo Seishima, Kohei Shigeta, Koji Okabayashi, Yuya Oaki

**Affiliations:** ^1^ Department of Applied Chemistry Faculty of Science and Technology Keio University 3‐14‐1 Hiyoshi, Kohoku‐ku Yokohama 223–8522 Japan; ^2^ Department of Surgery School of Medicine Keio University 35 Shinanomachi Shinjuku‐ku Tokyo 160–8582 Japan

**Keywords:** composites, conductive polymers, free volume, nanospace, polymers

## Abstract

Nanospace has been used as a specific field for syntheses and assemblies of molecules, polymers, and materials. Free volume space among polymer chains is related to their properties, such as permeation of gas and small molecules. However, the void has not been used as a functional nanospace in previous works. The present work shows synthesis of guest conductive polymers in free volume space of conventional synthetic resins and rubbers as a new nanospace. Vapor of heteroaromatic monomer and oxidative agent is diffused into the soft dynamic nanospace among the polymer chains under ambient pressure at low temperature. The oxidative polymerization provides the conductive polymers, such as polypyrrole (PPy), in the free volume space of poly(methyl methacrylate) (PMMA), polypropylene (PP), silicone rubber (SR), and polyurethane rubber (PU). The ratio of the free volume decreases with the infiltration of the conductive polymers. The composites exhibit the improved mechanical and gas barrier properties. The rubbers containing PPy are used as mechanical‐stress sensors with both the conductivity and flexibility. The free volume space of resins and rubbers can be used as a new dynamic nanospace for synthesis of functional polymer composites.

## Graphical abstract

A new functional nanospace has been found in conventional synthetic resins and rubbers. Free volume, nanoscale pore among polymer chains, is used as a reaction field for synthesis of conductive polymers. Functional composites with enhanced performances, such as mechanical, gas barrier, and conductive properties, are developed by the infiltration of the guest conjugated polymers in the free volume space.

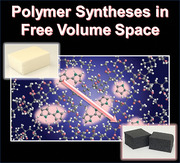

## 1 Introduction

**Figure 1:**
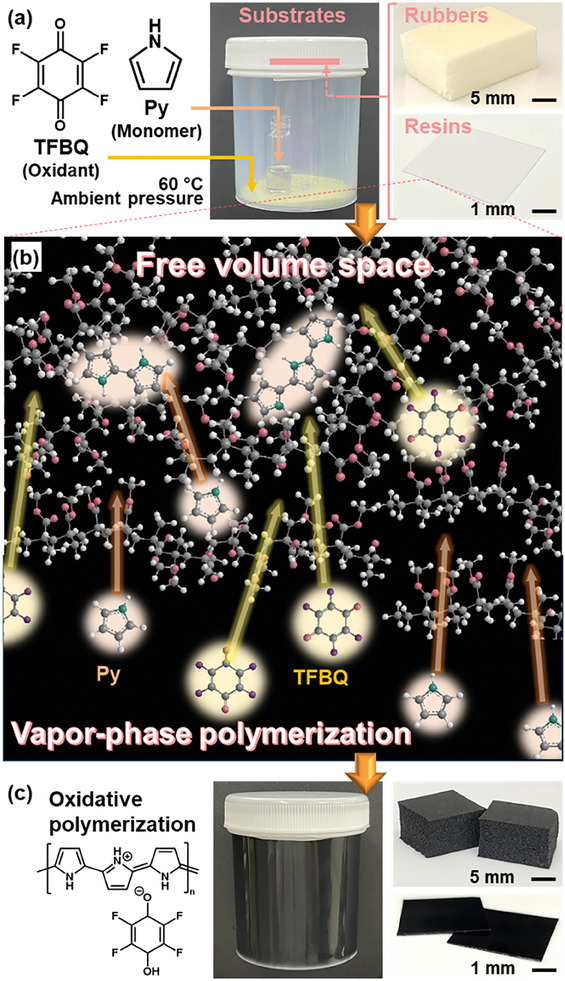
Synthesis of guest polymers in free volume space. a) Photographs of the experimental setup including pyrrole (Py) monomer, tetrafluoro‐1,4‐benzoquinone (TFBQ) oxidant, and substrates. b) Schematic illustration of the diffusion and vapor‐phase polymerization behavior in free volume space of poly(methyl methacrylate) (PMMA). c) Photographs of the sample bottle and substrates after the vapor‐phase synthesis of polypyrrole (PPy).

Nanoscale pore serves as not just a void but a versatile field for exploration of new materials and functions.^[^ ^1^, ^2^, ^3^, ^4^, ^5^, ^6^, ^7^, ^8^, ^9^, ^10^, ^11^, ^12^, ^13^, ^14^, ^15^, ^16^, ^17^ ^]^ Nanospace materials, such as zeolite,^[^ ^4^, ^5^ ^]^ layered materials,^[^ ^6^, ^7^ ^]^ micro‐ and mesoporous materials,^[^ ^8^, ^9^, ^10^ ^]^ metal‐organic frameworks (MOFs),^[^ ^11^, ^12^, ^13^, ^14^, ^15^ ^]^ and covalent organic frameworks (COFs),^[^ ^16^, ^17^ ^]^ accommodate guest ions and molecules for the storage, separation, assembly, and reaction. Synthesis of polymers using such nanospace has been studied to achieve new structures and properties.^[^ ^18^, ^19^, ^20^, ^21^, ^22^, ^23^, ^24^, ^25^ ^]^ For example, the confined space of MOF provided the polymers with the specific sequence and orientation.^[^ ^22^, ^23^ ^]^ The interspace between the nanocrystals was used for the morphology replication to the guest polymers.^[^ ^24^, ^25^ ^]^ In previous works, such polymer syntheses were studied in the static and rigid nanospace with the definite pore sizes measurable using gas adsorption.^[^ ^18^, ^19^, ^20^, ^21^, ^22^, ^23^ ^]^ Here we found that free volume space of typical synthetic resins and rubbers in the range of 0.5–0.8 nm, which is not measured by gas adsorption technique, can be used as a dynamic and soft nanospace for polymer synthesis (**Figure** **1**). The infiltration of the guest polymers enabled to tune and enhance the properties of the host polymers, such as mechanical strength, gas permeation, and conductivity.

Pores and channels of organic materials have been applied to separation, transportation, and permeation of target molecules and ions in liquid and gas phases.^[^ ^26^, ^27^, ^28^, ^29^, ^30^, ^31^, ^32^ ^]^ The size, morphology, and affinity of the nanospace were tuned by the molecular design.^[^ ^29^, ^30^, ^31^ ^]^ For example, reverse osmotic membrane is prepared to remove ions and molecules for production of purified water.^[^ ^32^ ^]^ Free volume in polymer materials has attracted interests because of the relevance to the properties.^[^ ^33^, ^34^, ^35^, ^36^, ^37^, ^38^, ^39^ ^]^ Small molecules in gas phase, such as water and oxygen, permeate free volume space.^[^ ^35^, ^37^ ^]^ The fluorescent molecules were introduced in the free volume space with the evaporation of solvents from the polymer solution to estimate the size and monitor the molecular motion.^[^ ^38^, ^39^ ^]^ The nanospace originating from the molecular and segment motions can be regarded as soft and dynamic compared with that of conventional host materials. However, the nanospace was not used for the reaction, assembly, and functionalization in previous works. The gas permeation properties inspire us to use the free volume space for synthesis of guest polymers from the monomer vapor. In the present work, the guest conductive polymers were synthesized by supplying vapor of heteroaromatic monomers and oxidative agents to the free volume space of conventional resins and rubbers (Figure 1a,b).

Conductive polymers having π‐conjugated main chain exhibit various properties, such as conductivity, redox activity, and luminescence.^[^ ^40^, ^41^, ^42^, ^43^, ^44^, ^45^, ^46^ ^]^ In general, conductive polymers are synthesized by oxidative or coupling polymerization of heteroaromatic monomers. However, the morphology control and processing are not easily achieved because of the insolubility and infusibility originating from the rigid conjugated main chain. The polymerization methods in the solution phase have been studied to control the size and morphology.^[^ ^46^ ^]^ Solvent‐free polymerization using monomer vapor has been developed for coating on the surfaces.^[^ ^47^, ^48^, ^49^, ^50^, ^51^, ^52^ ^]^ For example, oxidative chemical vapor deposition (o‐CVD) provides heteroaromatic polymers,^[^ ^53^ ^]^ such as polypyrrole (PPy)^[^ ^54^ ^]^ and thiophene derivatives.^[^ ^55^, ^56^ ^]^ Our group has developed a vapor‐phase polymerization method for the coating of conductive polymers at low temperature under ambient pressure.^[^ ^52^, ^57^, ^58^, ^59^ ^]^ The monomers, their activated species, and oxidative agents are supplied from vapor phase to the surface of substrates. The coating is obtained by the oxidative or coupling polymerization. In the present work, we found that the vapor‐phase synthesis provided conductive polymers, such as polypyrrole (PPy), not only on the surface but also inside conventional synthetic resins and rubbers (Figure 1c). As the monomers and oxidative agents in vapor phase permeated the free volume space (Figure 1b), the host‐guest polymer composites were obtained by the polymerization in the free volume space. The resultant composites showed the improved properties, such as mechanical strength and gas barrier. Moreover, PPy in a rubber exhibited both the stretchability and conductivity. The method can be applied to design functional polymer composites, such as reinforced materials and gas barrier films.

## 2 Results and Discussion

The conductive polymers were synthesized by supplying the monomer and oxidant from vapor phase in the presence of synthetic resins and rubbers at 60 °C under ambient pressure (Figure 1a). The detailed procedure was described in the Supporting Information. A small glass bottle (2 cm^3^) containing typically 10 mmol liquid heteroaromatic monomers, such as pyrrole (Py), was set in a polypropylene bottle as a reaction chamber (120 cm^3^). Powder of tetrafluoro‐1,4‐benzoquinone (TFBQ, 10 mmol) as a sublimable oxidative agent was spread over the bottom of the chamber. Substrates (4 cm × 4 cm × *t*) of synthetic resins and rubbers, namely poly(methyl methacrylate) (PMMA, *t* = 0.2 mm), polypropylene (PP, *t* = 1 mm), silicone rubber (SR, *t* = 0.5 mm), polyurethane rubber (PU, *t* = 6 mm), and polytetrafluoroethylene (PTFE, *t* = 1 mm), were attached on the inside of the screw cap using a double‐coated tape. After sealing, the reaction chamber was maintained in a drying oven at 60 °C under ambient pressure for 24 h. The substrate was collected and then vacuum‐dried at 60 °C for 48 h to remove the remaining monomer, oxidative agent, and oligomers.

**Figure 2:**
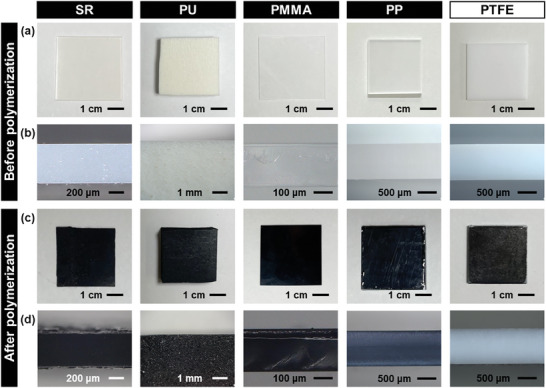
Photographs of SR, PU, PMMA, PP, and PTFE before a,b) and after c,d) the vapor‐phase synthesis of PPy. a,c) Surface images. b,d) Cross‐sectional optical microscopy images.

After the reaction, the black coating was observed on the inner wall of the reaction chamber (Figure 1c). The surface of the substrates was changed to black (**Figure** **2**a,c). Based on the cross‐sectional images (Figure 2b,d), SR, PU, PMMA, and PP exhibited the color changes not only on the surface but also inside the substrates. The color change was observed in the whole of the substrates for SR, PU, PMMA, and PP. In contrast, the coloration inside the substrate was not observed for PTFE (Figure 2d). In our previous works,^[^ ^52^, ^57^, ^58^, ^59^ ^]^ the surface coating of PPy was achieved on the surface of glass, metal, and inorganic salt. Here we found the coloration inside the synthetic resins and rubbers. The microscopy observation implies the formation of PPy in the free volume space of SR, PU, PMMA, and PP (Figure 1b).

### 2.1 Formation of PPy Inside the Resins and Rubbers

**Figure 3:**
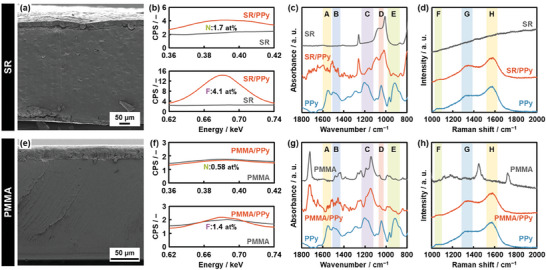
Structural analyses on the cross section of SR/PPy (a–d) and PMMA/PPy (e–h). a,e) SEM images. b,f) EDX spectra of the bare substrates (gray) and composites (orange) for nitrogen (upper panels) and fluorine (lower panels). c,g) FT‐IR spectra of the bare substrates (gray), composites (orange), and reference PPy (blue). d,h) Raman spectra of the bare substrates (gray), composites (orange), and reference PPy (blue).

The cross section of the color‐changed resins and rubbers was analyzed to study the formation of PPy. The results for SR and PMMA were shown in the main text (**Figure** **3**). The results for PU, PP, and PTFE were summarized in the Supporting Information (Figure S1, Supporting Information). Nitrogen (N) and fluorine (F) were detected on the cross section of the color‐changed SR and PMMA by energy‐dispersive X‐ray analysis (EDX) equipped with scanning electron microscopy (SEM) (Figure 3a,b,e,f; Figure S2, Supporting Information). The atomic concentration in the cross‐section was 1.7% of N and 4.1% of F for SR (area: 4.5 × 10^5^ µm^2^) and 0.58% of N and 1.4% of F for PMMA (area: 6.7 × 10^4^ µm^2^) (Table S1, Supporting Information). PU and PP showed the similar EDX results (Figure S1 and Table S1, Supporting Information). The appearance of these peaks indicates that PPy and its TFBQ dopant are contained in the rubber and resin. The additional EDX analysis on the magnified areas from the surface to the back side showed the peaks of N and F throughout the cross section (Figure S2 and Table S2, Supporting Information). In addition, N and F were detected by elemental analysis for organic compounds (Table S3, Supporting Information). These results indicate that the penetration of PPy inside the resins and rubbers, distinguished from the surface coating, was achieved for SR, PU, PMMA, and PP. The PPy concentration has a gradient in the depth direction (Figure S2 and Table S2, Supporting Information). The gradient originates from the slow diffusion of Py and TFBQ vapor inside the matrix polymer. As the free volume space is not fully occupied with PPy, the PPy formation is continued with the further diffusion of Py and TFBQ inside the matrix polymer. In addition, the diffusion is promoted by local dissolving and swelling the matrix polymer with inclusion of the monomer vapor. However, in this vapor polymerization system, the matrix polymers are not fully dissolved because the polymerization of Py rapidly proceeds with TFBQ. Therefore, PPy formed from the surface to bottom throughout the resins and rubbers. In contrast, only the surface coating was observed on PTFE (Figure 2; Figures S1 and S2 and Tables S1 and S2, Supporting Information).

The formation of PPy was studied by Fourier‐transform infrared (FT‐IR) and Raman spectroscopies (Figure 3c,d,g,h; Figure S1, Supporting Information). The product on the surface of the TFBQ crystals in the same chamber was collected to prepare the reference PPy. Then, PPy was obtained by the dissolution of the remaining TFBQ and vacuum drying. FT‐IR spectrum was measured on the color‐changed cross sections by attenuated total reflection (ATR) (Figure 3c,g). The weak absorption bands characteristic of PPy were observed in addition to the spectra of the substrate themselves: C═C and C─C stretching vibrations in the ring (A), C═C and C─N stretching vibration in the ring (B), complex C═C, C─C, and C─N stretching vibrations in the ring (C), C─H in‐plane and out‐of‐plane bending and ring deformation vibrations (D), and ring bending vibration (E).^[^ ^60^, ^61^ ^]^ The more distinct changes were observed in the Raman spectra. The cross section showed the peaks characteristic of PPy (Figure 3d,h): C─H in‐plane bending vibration (F), C─N stretching vibration (G), and C═C stretching vibration (H).^[^ ^60^, ^62^ ^]^ Whereas the similar spectroscopic changes were observed for PU and PP (Figure S1, Supporting Information), no such changes were observed for PTFE. These spectroscopic analyses on the cross section support the formation of PPy inside the resins and rubbers except PTFE. The conjugation length of PPy formed in the free volume space was similar to that of commercially available PPy in the powdered state (Figure S3, Supporting Information). The fact implies that the polymerization providing the conductive polymer with the sufficient conjugation length is achieved even in the nanospace.

**Table** **1** summarizes the PPy proportions (wt%) in the resins and rubbers estimated from the EDX quantification and CHN elemental analyses. The EDX spectra for the quantification were obtained on the whole of the cross section from the surface to bottom (Table 1; Table S1, Supporting Information). EDX analysis was mainly used for the qualitative study of the PPy distribution and rough estimation of the PPy content. On the other hand, the more accurate quantification was performed by CHN elemental analysis. The PPy proportion was calculated from the differences in the atomic concentration of N originating from PPy before and after the polymerization (Tables S1 and S3, Supporting Information). For example, EDX and elemental analyses of SR/PPy indicate the PPy proportion 4.26% and 4.78%, respectively. The PPy proportions calculated from the EDX and elemental analyses are consistent with each other except PU (Table 1).

**Table 1:** Decrease in the free volume and PPy proportions.

Sample	Lifetime *τ*_3_ / ns	Estimated pore radius *r*_3_ / nm	Intensity *I*_3_ / %	Free volume fraction *f* / vol%	Δ*I*_3_ / %	PPy / wt% [EDX]	PPy / wt% [Elemental analysis]
SR	3.27	0.382	31.9	13.4	−8.6	4.26	4.78
SR/PPy	3.38	0.389	23.3	10.3			
PU	2.28	0.310	21.3	4.78	−6.7	3.58 ^a^ ^)^	0.77 ^a^ ^)^
PU/PPy	2.17	0.300	14.6	2.97			
PMMA	1.82	0.268	27.5	3.99	−3.8	2.35	1.19
PMMA/PPy	1.83	0.270	23.7	3.5			
PP	2.15	0.299	23.2	4.68	−1.4	0.989	0.77
PP/PPy	2.15	0.299	21.8	4.39			
PTFE	3.41	0.390	23.7	10.6	−0.6	−0.126^b^ ^)^	– ^c^ ^)^
PTFE/PPy	3.54	0.398	23.1	11.0			

^a)^ The accurate proportion was not calculated by the increment of nitrogen content because the original PU contained nitrogen.

^b)^ The negative value indicates that PPy is not formed in PTFE.

^c)^ The elemental analysis was not carried out.

### 2.2 Decrease in Free Volume Space

The free volume space decreased with the penetration of PPy (Table 1 and Figure S4, Supporting Information). Positron annihilation lifetime spectroscopy (PALS) was used to analyze the changes in the free volume space of the host polymers. Free volume of polymers is analyzed based on the annihilation of the positronium trapped in void and electron on the wall.^[^ ^63^, ^64^ ^]^ In PALS analysis, the annihilation with forming the positronium is preferentially observed in the low electron density regions, such as voids and free volume.^[^ ^35^ ^]^ In other words, the longer lifetime is achieved by the annihilation in the pore space. The effect of the pore wall and material itself on the annihilation can be negligible to estimate the lifetime in the pore space. In general, the lifetime of the third content (*τ*_3_: 1∼4 ns) in the attenuation curve corresponds to the annihilation in the free volume space of polymers (Figure S4, Supporting Information). Here *τ*_3_ is estimated from the lifetime curve by the approximation to log‐normal distribution. As the radius of the free volume (*r*_3_) is calculated from *τ*_3_ with fitting to Tao‐Eldrup equation on the assumption of the spherical pore,^[^ ^65^, ^66^ ^]^ the free‐volume fraction (*f*) is estimated from the relative intensity (*I*_3_) and total free volume (*V*_3_ (= 4π*r*_3_^3^ / 3)). The detailed method was described in the Supporting Information.^[^ ^67^, ^68^ ^]^

*τ*_3_ for all the samples was observed in the range of 1 and 4 ns (Table 1). The pore size of the free volume space (2*r*_3_) was estimated to be in the range of 2*r*_3_ = 0.5–0.8 nm. As the size of the molecules is calculated to be 0.43 nm for Py and 0.55 nm for TFBQ, the monomer and oxidant molecules permeate the free volume space and diffuse inside the resins and rubbers with the molecular and segment motions. Whereas *I*_3_ and *f* decreased after the polymerization, *τ*_3_ and *r*_3_ were not distinctly changed. A distinct decrease in the pore radius (*r*_3_) of free volume was not observed after the deposition of PPy (Table 1). The concentration of PPy in the matrix polymer is low to induce the decrease in the pore radius. The fact implies that only a small amount of free volume space in matrix polymer is filled with PPy. The differences in *I*_3_ between the original substrate (*I*_3,o_) and composite (*I*_3,c_) (Δ*I*_3_% = *I*_3,c_ − *I*_3,o_ < 0) corresponds to the infiltration of PPy with the decrease in the free volume space. The Δ*I*_3_ and PPy proportions estimated from the EDX and elemental analyses are consistent with each other for SR, PU, PMMA, and PP (Table 1). In contrast, PTFE showed no significant changes in the Δ*I*_3_. As the reference, a commercial PPy powder and that formed on the TFBQ crystal were compressed to obtain the pellets. *I*_3_ were 0.39% (τ_3_ = 2.38 ns) for the commercial PPy and 1.26% (τ_3_ = 1.37 ns) for the synthesized one. The results indicate that PPy itself has no free volume space. The free volume space is not generated from the stacking of the rigid rod‐like PPy molecules.

In some samples, the atomic concentration of F is higher than that assuming the fully doped state (Table S3, Supporting Information), even though it is not easy to estimate the real doping level, i.e., the actual concentration of TFBQ with the doped state. When only Py monomer or TFBQ vapor was diffused into SR, a decrease in free volume with decreasing Δ*I*_3_ was observed (Table S4, Supporting Information). The fact implies that free TFBQ molecules that are not doped in PPy is introduced in the matrix polymer. The TFBQ‐doped PPy is synthesized in the free volume space through the diffusion of the monomer and oxidative agent and subsequent oxidative polymerization. The differences in the PPy proportions are determined by the permeation behavior of Py and TFBQ in the resins and rubbers. Moreover, the penetration of the conductive polymer in these resins and rubbers were not observed by the polymerization in the solution phase (Figure S5 and Table S5, Supporting Information). The Py monomer and TFBQ oxidative agent are not penetrated into the polymer substrates from the liquid phase. Molecules are interacted with each other in the condensed state, such as neat liquid of Py. In the diluted solution, such as the TFBQ solution, molecules are solvated by the solvent molecules. As the isolated states of molecules are not formed in the condensed states, Py and TFBQ are not easily penetrated into the polymer substrates. In contrast, the isolated Py and TFBQ molecules in vapor phase can be penetrated into the free volume space more easily.

**Figure 4:**
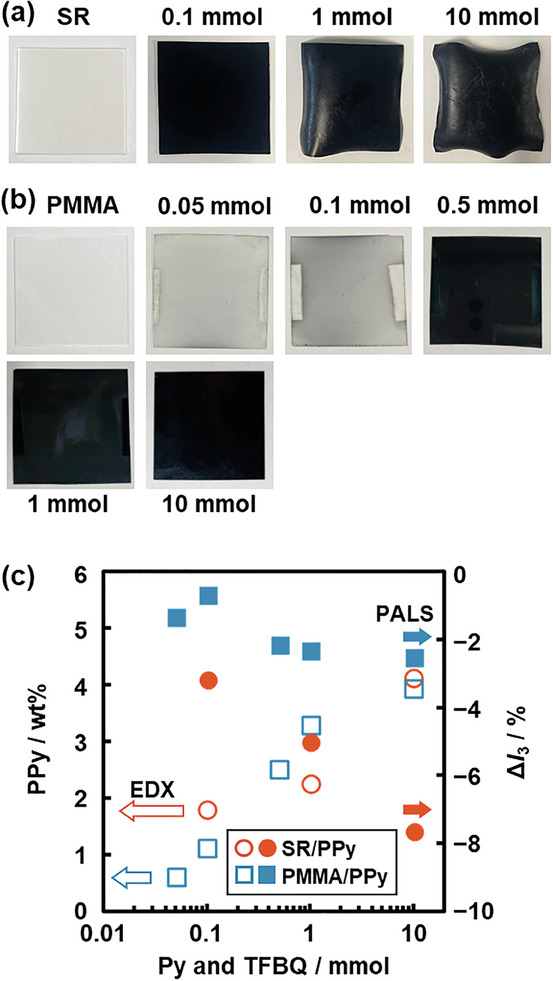
Synthesis of SR/PPy and PMMA/PPy with changes in the initial amount of Py and TFBQ (*N* = 3). a,b) Photographs of the bare SR a) and PMMA b) substrates (left panel) and their composites with changes in the initial amount of Py and TFBQ (right panels). c) Relationship between the initial amount of Py and TFBQ (horizontal axis) and PPy proportion (open symbols, left axis) and Δ*I*_3_ corresponding to the decrease in the free volume calculated from the PALS analysis (filled symbols, right axis).

The PPy proportions were controlled by changes in the initial amounts of Py monomer and TFBQ oxidant (**Figure** **4** and Table S6, Supporting Information). The SR/PPy and PMMA/PPy were prepared in the different conditions with a decrease in the initial amounts of Py and TFBQ (both 10 mmol in the original condition) three times (the number of samples: *N* = 3) to ensure the reproducibility. The deformation of the elastic SR substrate was suppressed with a decrease in the initial amounts of Py and TFBQ (Figure 4a). The coloration of PMMA substrate reduced with the Py and TFBQ amounts lower than 0.5 mmol (Figure 4b). The average PPy proportions (wt.%) estimated from the EDX analysis increased with an increase in the initial monomer amounts (the left axis and open symbols in Figure 4c). Δ*I*_3_ estimated from the PALS analyses decreased with increasing the initial amounts (the right axis and filled symbols in Figure 4c). The results indicate that the PPy proportions in the free volume space were controlled by the initial amounts of the monomer and oxidative agent. Although the PPy concentration increased (Figure 4c), the free‐volume space is not fully occupied with PPy. As the diffusion rate of Py and TFBQ in the matrix polymers is slow, it is not easy to achieve the full occupation of the free volume under the present conditions.

The similar composites based on SR were obtained using the different monomers, namely 1‐methylpyrrole (1‐Me‐Py), 1‐ethylpyrrole (1‐Et‐Py), 1‐aminopylpyrrole (1‐Am‐Py), and 3,4‐ethylenedioxithiophene (EDOT) (Figures S6 and S7 and Table S7, Supporting Information). In this manner, the conductive polymers were synthesized by the vapor‐phase method in the free volume space of synthetic resins and rubbers. If the monomers and related reagents are supplied from vapor phase, the other guest polymers can be synthesized in the free volume space of resins and rubbers. The fraction of free volume and coefficient of gas permeability have effects on the polymerization rate and proportion of the guest polymers. The polymerization behavior is also different for the size and structure of monomers. Free volume space can be applied to as a functional nanospace for syntheses and assemblies of molecules and polymers.

### 2.3 Potential Applications of the PPy‐Contained Composites in the Free Volume Space

**Figure 5:**
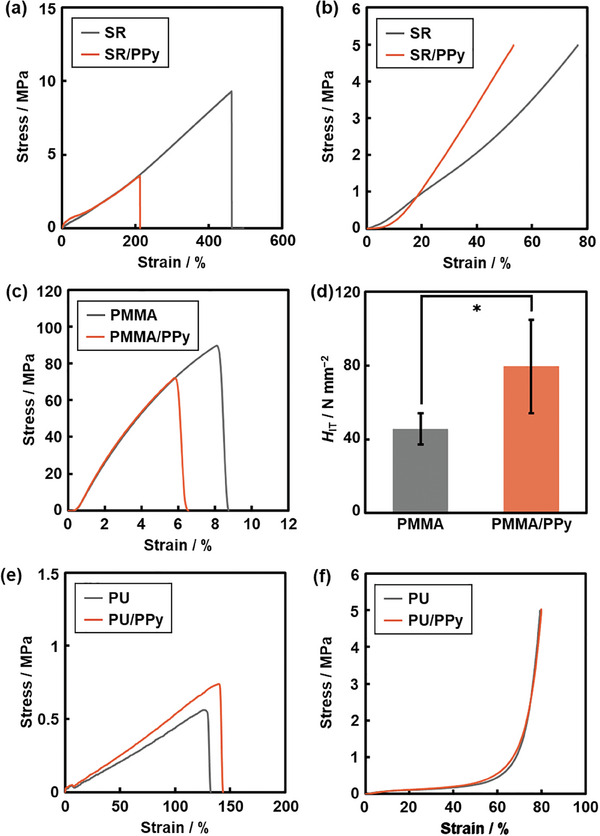
Mechanical properties of SR/PPy a,b), PMMA/PPy c,d), and PU/PPy e,f) compared with those of the bare substrate. (a,c,e) Stress‐strain curves of the tensile tests (*N* = 5). b,f) Stress–strain curves of the compression tests (*N* = 5). d) Indentation hardness (*N* = 10, error bars: 95% confidence interval, *: *p* < 0.05). The representative data were displayed in these panels. All the data are listed in Figure S8 (Supporting Information).

A new polymer composite was obtained with the infiltration of the conjugated polymer as a molecular filler in the free volume space of the matrix polymer. Here we studied a couple of the potential applications. The mechanical properties of SR/PPy, PU/PPy, and PMMA/PPy were changed compared with those of the pristine substrates (**Figure** **5**). These samples were prepared at 10 mmol initial amounts of Py and TFBQ. In the stress‐strain curve of the tensile test, the original SR showed the tensile strength 8.97 ± 1.2 MPa and break elongation 476 ± 97% (*N* = 5, the gray line in Figure 5a; Figure S8, Supporting Information). The tensile strength and break elongation decreased to 3.69 ± 0.40 MPa and 209 ± 36% for SR/PPy, respectively (*N* = 5, the orange line in Figure 5a; Figure S8, Supporting Information). In the compression test, the strain of SR/PPy was smaller than that of SR under the same applied stress (*N* = 5, Figure 5b; Figure S8, Supporting Information). Whereas the original PMMA showed the tensile strength 95.3 ± 15 MPa and break elongation 8.5 ± 1.8%, these values decreased to 74.8 ± 6.9 MPa and 6.0 ± 1.0% for PMMA/PPy, respectively (*N* = 5, Figure 5c; Figure S8, Supporting Information). The Martens hardness (*H*_IT_) was 45.5 ± 8.5 N mm^−2^ for PMMA and 79.5 ± 25.3 N mm^−2^ for PMMA/PPy by an indentation method (ISO 14 577) (*N* = 10, Figure 5d). The incorporation of PPy in the free volume of SR and PMMA induces the increase in the hardness and decrease in the elastic properties. The tensile strength 0.57 ± 0.06 MPa and break elongation 125 ± 11% for PU increased to 0.78 ± 0.12 MPa and 147 ± 9.7% for PU/PPy, respectively (*N* = 5, Figure 5e; Figure S8, Supporting Information). The compression behavior was not changed for PU and PU/PPy (Figure 5f). As the PPy proportion of PU/PPy is lower than that of the SR/PPy and PMMA/PPy, the stretching behavior originating from the soft segment of PU is preserved even after the infiltration of PPy. In general, carbon nanomaterials, such as fibers and sheets, are used for the reinforcement of the polymer materials.^[^ ^69^ ^]^ The mechanical properties of polymer materials are tuned by the bonding and entangled states of the polymer chains and networks.^[^ ^70^, ^71^ ^]^ The present work indicates that the guest polymers in free volume space can be used as a new filler in molecular level for tuning the mechanical properties of the host polymers.

Free volume space involves the permeation properties of small molecules in gas phase.^[^ ^35^, ^36^, ^37^ ^]^ The gas permeation rate decreased with the infiltration of the guest polymer in the free volume space. The permeation rate of water vapor was 14.1 ± 0.11 g m^−2^ day^−1^ for PMMA and 11.3 ± 0.18 g m^−2^ day^−1^ for PMMA/PPy in the repetitive tests to the same samples six times (Figure S9, Supporting Information). Moreover, the gas barrier performance was improved with an increase in the amount of the incorporated PPy (Figure S9, Supporting Information). PMMA/PPy inhibits the permeation of water vapor with infiltration of PPy in the free volume. In previous works, the improved gas barrier performances were achieved by the dispersion of inorganic clays in the matrix polymers.^[^ ^72^ ^]^ The present results imply that vapor‐phase polymerization in free volume space can be used as a new method to control gas barrier performances of polymer materials.

### 2.4 Mechanoresponsive Conductive Rubbers

**Figure 6:**
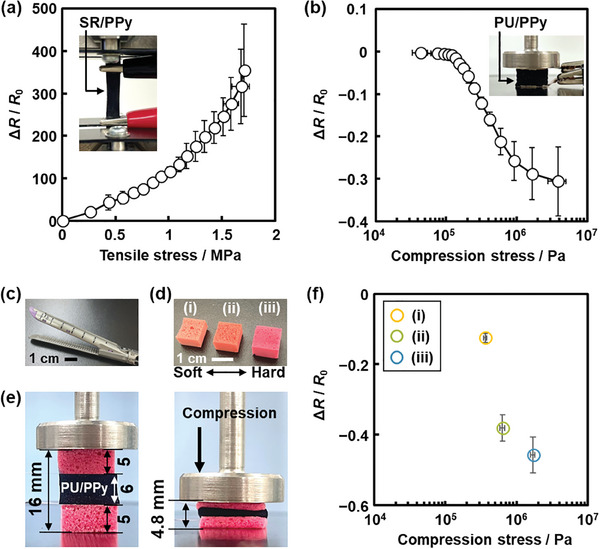
Application of the SR/PPy and PU/PPy conductive rubbers for mechano sensing. a) Relationship between the tensile stress and Δ*R* / *R*_0_ of SR/PPy (*N* = 3). b) Relationship between the compression stress and Δ*R* / *R*_0_ of PU/PPy (*N* = 3). c) Photograph of an automated linear stapler. d) Softness models (i, soft), (ii, medium), and (iii, hard) of intestinal tracts. e) Photographs of PU/PPy set between the two softness models before (left) and after (right) the application of the compression stress to reach the strain 70%. f) Relationship between the Δ*R* / *R*_0_ and measured compression stress of the softness models using the PU/PPy device (*N* = 3).

SR and PU containing PPy were used as conductive rubbers exhibiting both the elastic and conductive properties (**Figure** **6**). In recent years, a variety of soft and flexible conductive materials have been studied using the composites containing conductive nanomaterials, such as carbon nanoparticles, metal nanowires, and conductive polymers.^[^ ^73^, ^74^ ^]^ In the present work, the host matrix rubber was transformed to the conductive materials with the introduction of PPy in the free volume space. In general, it is not easy to design the molecules with mechanical softness and electrical conductivity because introduction of rigid π‐conjugated moiety for conductivity lowers softness of the materials. Here the PPy proportion in SR was lowered to enhance the stretching properties with decreasing the initial amounts of Py and TFBQ (10 to 1 mmol) (Figure S10, Supporting Information). The initial conductivity (*σ*) was (2.46 ± 0.81) × 10^−7^ S cm^−1^ for SR/PPy and (9.55 ± 0.57) × 10^−5^ S cm^−1^ for PU/PPy (*N* = 3), whereas *σ* of SR and PU before the polymerization was not measured because of the overload. The pellet of the reference PPy powder formed on the TFBQ crystal showed *σ* = (8.33 ± 0.45) × 10^−2^ S cm^−1^. The conductivity of SR/PPy and PU/PPy was lower than that of the pure PPy samples. The conductive pathway is not fully expanded in SR and PU. On the other hand, it is noteworthy that the conductivity is achieved even at PPy concentration lower than 5%. As the sample was deformed with the application of the mechanical stresses, the responsivity Δ*R* (= *R* − *R*_0_) / *R*_0_ was used instead of *σ* as a metric for the conductivity, where *R*_0_ is the resistance in the initial state and *R* is the resistance in the stressed state. SR/PPy showed an increase in Δ*R* / *R*_0_ with increasing the applied tensile stress smaller than 1.71 MPa (*N* = 3, Figure 6a; Figure S11, Supporting Information). A decrease in Δ*R* / *R*_0_ of PU/PPy was observed with the application of the compression stress smaller than 3.88 MPa (Figure 6b; Figure S11, Supporting Information). The overlapped state of the π‐conjugated PPy molecules in the free volume space is changed by the applied mechanical stresses. Therefore, the conductive and elastic composites show the mechanoresponsive changes in Δ*R* / *R*_0_.

A potential medical application was demonstrated using the mechanoresponsive PU/PPy (Figure 6c–f). The softness of the intestinal tracts was measured using the model rubbers and PU/PPy. When cancer tissue in intestinal tract is removed, surgeons use stapling devices for suture and anastomosis. The tract is compressed, stapled, and cut using automated staplers (Figure 6c). The selection of an appropriate stapling device depending on the thickness and softness of individual tracts is needed to achieve the safe suture and anastomosis because the failure causes serious complications. However, in the clinical site, the softness is not measured using sensors but estimated based on the experience of surgeons. Here the three softness models of the tracts, namely soft i), medium ii), and hard iii) (Figure 6d), were differentiated by Δ*R* / *R*_0_ using the PU/PPy conductive rubber (Figure 6e). The model tracts i–iii) showed the different stress‐strain curves in the compression test (Figure S12, Supporting Information). Prior to the demonstration, the conductivity and responsivity of PU/PPy was improved with increasing the proportion of PPy in the composite (Figure S13, Supporting Information). The PU/PPy device set between the two same models was compressed to reach the strain 70% (Figure 6e). The models i–iii) showed Δ*R* / *R*_0_ −0.125 ± 0.013, −0.381 ± 0.037, and −0.457 ± 0.051 (*N* = 3, Figure 6f), respectively. The differences in the softness were measured by Δ*R* / *R*_0_ using the PU/PPy device. In this manner, the conductive rubber device was applied to measure the mechanical stress and hardness. Such conductive rubber can be integrated in a sensing system of mechanical stresses. The sensor can be applied to measure the softness of the organs for selection of appropriate operating devices.

## 3 Conclusions

Free volume space of synthetic resins and rubbers was used as a soft and dynamic nanospace for synthesis of guest polymers. Gas permeation properties of free volume inspired us to utilize vapor‐phase synthesis of conductive polymers. Whereas PMMA, PP, SR, and PU formed the composites with the infiltration of PPy in the free volume space, only the coating was observed on the surface of PTFE. The mechanical and gas permeation properties were changed with the introduction of PPy in the free volume space. The soft conductive rubbers based on SR and PU were applied to mechanoresponsive sensing materials. The vapor‐phase synthesis in free volume space can be applied to the other combination of the host and guest polymers. New functional polymer composites can be obtained using the free‐volume space as a soft and dynamic nanospace.

## Conflict of Interest

The authors declare no conflict of interest.

## Supporting information



Supporting Information

## Data Availability

The data that support the findings of this study are available in the supplementary material of this article.
