# Supplementary material for: Free Volume Space of Polymers as a New Functional Nanospace: Synthesis of Guest Polymers
Source: Macromol Rapid Commun. 2025 Jan 30;46(8):2400980. doi: 10.1002/marc.202400980 (PMC12004908; doi:10.1002/marc.202400980)
Supplement: Supplementary file 1 — Supporting Information [file MARC-46-2400980-s001.pdf]

Macromolecular Rapid Communications

Supporting Information

for *Macromol. Rapid Commun.*, DOI 10.1002/marc.202400980

Free Volume Space of Polymers as a New Functional Nanospace: Synthesis of Guest Polymers

*Sayaka Hirai, Tomoki Sakuma, Yuki Tokura, Hiroaki Imai, Ryo Seishima, Kohei Shigeta, Koji Okabayashi and Yuya Oaki**

Supporting Information

Free Volume Space of Polymers as A New Functional Nanospace: Synthesis of Guest Polymers

Sayaka Hirai,[a] Tomoki Sakuma,[a] Yuki Tokura,[a] Hiroaki Imai,[a] Ryo Seishima,[b] Kohei Shigeta,[b] Koji Okabayashi,[b] Yuya Oaki*,[a]

[a] Department of Applied Chemistry, Faculty of Science and Technology, Keio University, 3-14-1 Hiyoshi, Kohoku-ku, Yokohama 223-8522, Japan.

[b] Department of Surgery, School of Medicine, Keio University, 35 Shinanomachi, Shinjuku-ku, Tokyo 160-8582

*E-mail: oakiyuya@applc.keio.ac.jp

Contents

Experimental methods	S-2
Structural analyses of PU/PPy, PP/PPy, and PTFE/PPy (Figure S1)	S-4
Quantification by EDX analysis (Table S1)	S-5
EDX analysis on the magnified area of the cross section (Figure S2 and Table S2)	S-6
Quantification by CHN and halogen elemental analysis (Table S3)	S-8
UV-Vis-NIR spectra (Figure S3)	S-9
Lifetime curves of PALS and their analysis (Figure S4)	S-10
Penetration of Py and TFBQ (Table S4)	S-12
Solution-phase polymerization as a reference (Figure S5 and Table S5)	S-13
Control of the PPy proportions in the composites (Table S6)	S-15
Syntheses of the other conductive polymers (Figures S6 and S7 and Table S7)	S-16
Reproducibility of the mechanical properties (Figure S8)	S-19
Permeation rate of water vapor (Figure S9)	S-20
Mechanical properties of SR/PPy (Figure S10)	S-21
Responsivity of SR/PPy and PU/PPy (Figure S11)	S-22
Stress-strain curves of the softness models (Figure S12)	S-23
PU/PPy sample for sensing compression stresses (Figure S13)	S-24

Experimental methods

Vapor-phase synthesis of PPy. Vapor-phase synthesis of the conductive polymers was referred to the procedure in our previous report.[57–59,S1] The following heteroaromatic monomer in liquid state was set in a glass bottle (2 cm^3, 12 mm in diameter and 35 mm in height): Py (TCI 99.0 %), EDOT (TCI 99.0 %), 1-Me-Py (TCI 99.0 %), 1-Et-Py (TCI 98.0 %), and 1-Am-Py (TCI 98.0 %). The Py amount was set at 10 mmol as the standard condition. The bottle was put in a polypropylene vial as a reaction chamber (120 cm^3, 57 mm in diameter and 72 mm in height). Powder of TFBQ (TCI 98.0 %) equimolar to Py was spread over the bottom of the chamber. The following substrates (4 cm × 4 cm × t mm) of synthetic resins and rubbers were attached on the inside of the screw cap using a double-coated tape: poly(methyl methacrylate) (PMMA, t = 0.2 mm), polypropylene (PP, t = 1 mm), polytetrafluoroethylene (PTFE, t = 1 mm), silicone rubber (SR, t = 0.5 mm), and polyurethane rubber (PU, t = 6 mm). After sealing, the reaction chamber was maintained in a drying oven at 60 °C under ambient pressure for 24 h. The substrate was collected and vacuum-dried at 60 °C for 48 h to remove the remaining monomer, oxidative agent, and oligomers. The resultant powder at the bottom of the chamber was rinsed with acetone to dissolve TFBQ and then vacuum-dried at 60 °C for 48 h. The collected PPy was used as the reference sample to the composite samples.

The initial amounts of Py and TFBQ were set at 0.1, 1, and 10 mmol for SR and 0.05, 0.1, 0.5, 1, and 10 mmol for PMMA to control the PPy proportions (Figure 4). In these cases, the equimolar TFBQ was spread over the bottom of the chamber. The other procedure was the same as that of the standard condition.

The polymerization of the other monomers, such as 1-Me-Py, 1-Et-Py, 1-Am-Py, and EDOT, was performed using SR by the alternative exposure to the monomers and TFBQ. First, SR was set in the chamber only containing 5 mmol Py for 24 h at 60 °C under ambient pressure. Then, the resultant Py-introduced SR without drying was set in the chamber only containing 5 mmol TFBQ for 24 h at 60 °C under ambient pressure.

Structural characterization. The substrates were cut using a guillotine-type machine to expose the cross section. The sample color on the surface and cross-section was observed by a smart phone (iphone) and optical microscopy (Keyence VHX-970F). The morphology and composition were analyzed using scanning electron microscopy (SEM, Carl-Zeiss Merin-VP compact) equipped with EDX (Bruker Quantax EDS). FT-IR (Jasco FT/IR-4100) spectra were collected on the cross section of the samples by ATR method. Raman spectra were also measured on the cross section (Renishaw InVia Raman, excitation light: 632 nm). The free volume fraction was measured using PALS (Toyo Seiko, PSA Type L-II) with Na-22 as positron source. A program IPALM was used to estimate the lifetimes and their relative intensities from the spectra. UV-Vis-NIR spectra were measured using a spectrophotometer (Jasco V-670).

Mechanical properties. The sample was cut into a rectangular shape 40 mm × 4 mm in size. The sample was loaded to a tester (Shimadzu EZ-LX) using the jigs with preserving the stretching area 15 mm × 4 mm. The tensile stress was applied to PMMA/PPy, SR/PPy, and PU/PPy at the rate 100 mm min^{-1}. The stress-strain curves were obtained. The compression stress was applied to SR/PPy and PU/PPy using the same tester. The sample 10 mm × 10 mm in size was compressed using a metallic probe 15 mm in diameter at the rate 10 mm min^{-1}. Martens hardness was measured by dynamic ultra-micro hardness tester (Shimadzu DUH-211S). The stress was applied at 13.324 mN s^{-1} using Berkovich-type triangular pyramid indenter with the edge angle 115 °.

Gas permeation properties. The permeation rate of water vapor was measured for PMMA and PMMA/PPy (Mocon AQUATRAN). One side of the sample was exposed to water vapor atmosphere, and the opposite side was maintained at 0 % RH. The amount of water vapor permeating through the sample was measured by the differences in the partial pressure. The sample was set under 40 °C and 90 % RH to measure the permeability of water vapor.

Conductive properties. The SR/PPy (40 mm × 4 mm × 0.5 mm) sample was prepared by the same methods using 1 mmol of Py and equimolar TFBQ. The SR/PPy film was set between the jigs with the distance 15 mm (Figure 6a). After the probe was attached to the film with the distance 10 mm, the conductivity was measured under the application of the tensile stress at the rate 1 mm s^{-1}. The PU/PPy sample was prepared at the standard condition (10 mmol Py and TFBQ). Copper foils as electrode were attached both the sides of PU/PPy using conductive silver paste (Figure 6b). The conductivity was measured using a digital multimeter (Keysight Truevolt Digital Multimeter) under the application of the compression stress at the rate 10 mm min^{-1}.

The PU/PPy (10 mm × 10 mm × 6 mm) samples were applied to detect the softness of the model tracts. The sample was prepared by the alternative exposure to Py and equimolar TFBQ vapor to form the homogeneous composites. PU was set in the chamber only containing 10 mmol Py for 24 h at 60 °C under ambient pressure. Then, the Py-introduced rubber without drying was set in the chamber only containing 10 mmol TFBQ for 24 h at 60 °C under ambient pressure. This cycle exposing Py and TFBQ vapor was repeated two times. The substrates were dried at 60 °C for 48 h under vacuum condition. After copper foils were attached on the top and bottom sides of PU/PPy, the rubber was sandwiched between the same softness models 5 mm in thickness, as shown in Figure 6e. The PU/PPy and softness models were compressed with strain 70 % using a tester and the resistance (*R*) was measured.

Additional Reference

S1. K. Sato, H. Imai, Y. Oaki, Y. *ACS Appl. Nano Mater.* **2018**, *1*, 4218.

Structural analyses of PU/PPy, PP/PPy, and PTFE/PPy

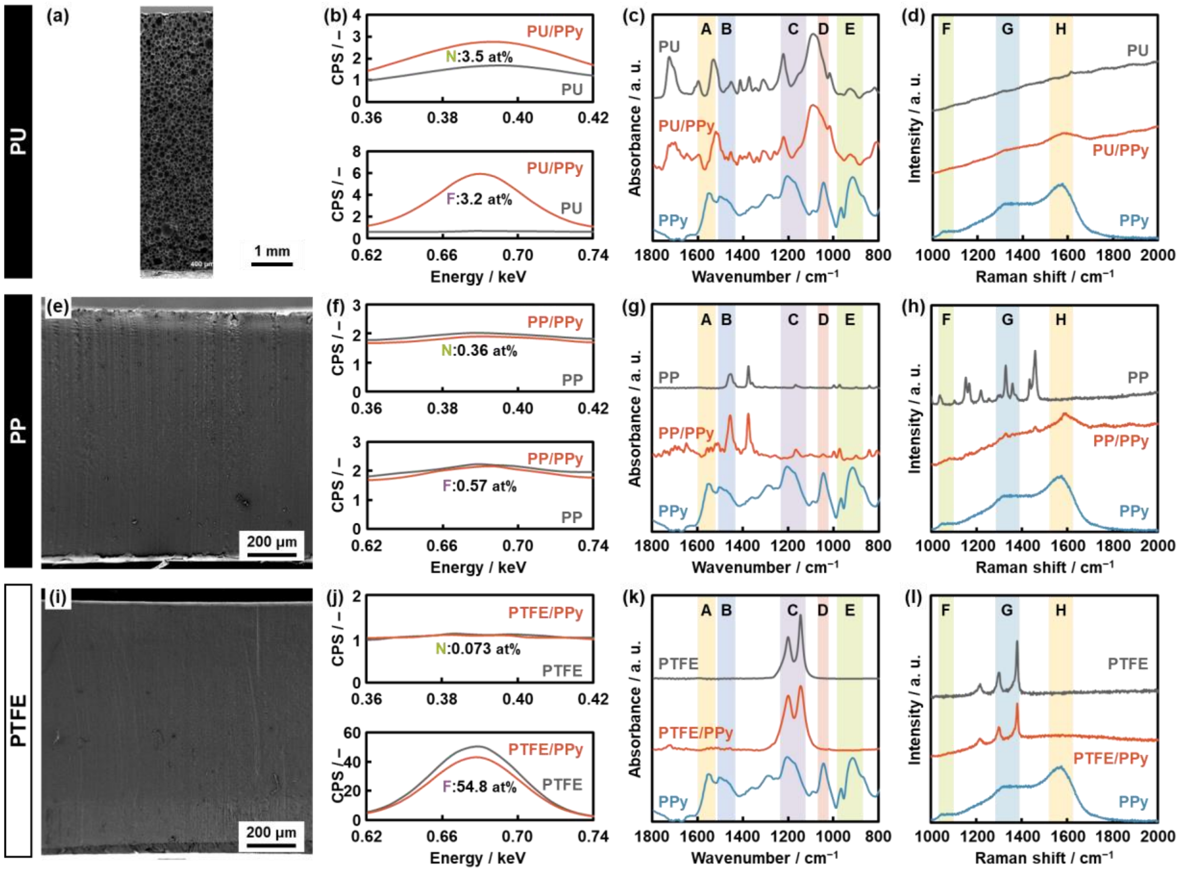

Figure S1. Structural analyses on the cross section of PU/PPy (a–d), PP/PPy (e–h), and PTFE/PPy (i–l). (a,e,i) SEM images. (b,f,j) EDX spectra of the bare substrates (gray) and composites (orange). (c,g,k) FT-IR spectra of the bare substrates (gray), composites (orange), and reference PPy (blue). (d,h,l) Raman spectra of the bare substrates (gray), composites (orange), and PPy (blue).

Based on the SEM-EDX, FT-IR, and Raman analyses, PPy was contained in the PU/PPy and PP/PPy substrates (Figure S1a–h). The detailed peak assignments of FT-IR and Raman were shown in the main text. In contrast, PPy was not introduced in PTFE (Figure S1i–l). In addition to the cross-sectional image (Figure 2), no peaks characteristic of PPy were observed for PTFE in the FT-IR and Raman spectra (Figure S1k,l). These results were supported by PALS analyses in Table 1.

Quantification by EDX analysis

Table S1. Quantification of N and F in the substrates and composites.

Substrates	N / atomic %	F / atomic %	PPy / wt %
SR	0.51	0.059	–
SR/PPy	1.67	4.13	4.26
PU	2.72	0.082	–
PU/PPy	3.49	3.18	3.58
PMMA	0.074	0.30	–
PMMA/PPy	0.58	1.36	2.35
PP	0.15	0.34	–
PP/PPy	0.36	0.47	0.989
PTFE	0.11	57.2	–
PTFE/PPy	0.073	54.8	−0.126

The EDX spectra were collected in the cross sections throughout the surface to the back sides, as shown in Figure 3a,e and Figure S1a,e,i. The PPy proportions were calculated from the EDX quantification results by the following procedure. The increment of nitrogen proportion (Δx_N / –) was calculated using (Eq. S1), where $x_{N,c}$ is atomic ratio of nitrogen in the composite and $x_{N,s}$ is that in the original substrate.

$$\Delta x_N = x_{N,c} - x_{N,s} \quad \text{... (Eq. S1)}$$

The weight ratio of carbon ($W_{C,c}$) in the composite was calculated using (Eq. S2), where $x_{X,c}$ is the atomic ratio of each element (X = C, N, O, F, Si) in the composites by the EDX quantification results.

$$W_{C,c} = \frac{x_{C,c} \times 12.01}{x_{C,c} \times 12.01 + x_{N,c} \times 14.01 + x_{O,c} \times 16.00 + x_{F,c} \times 19.00 + x_{Si,c} \times 28.09} \quad \text{... (Eq. S2)}$$

The weight ratio of PPy ($W_{PPy,c}$) in the composite was calculated using (Eq. S3) based on the molecular structure of Py unit, where $W_{N,c}$ is the weight ratio of N.

$$W_{PPy,c} = W_{C,c} \times \left(\frac{\Delta x_N}{x_{C,c}}\right) \times 4 + W_{N,c} \times \left(\frac{\Delta x_N}{x_{N,c}}\right) \quad \text{... (Eq. S3)}$$

The calculated $W_{PPy,c}$ (wt%) was summarized in Table 1.

EDX analysis on the magnified area of the cross section

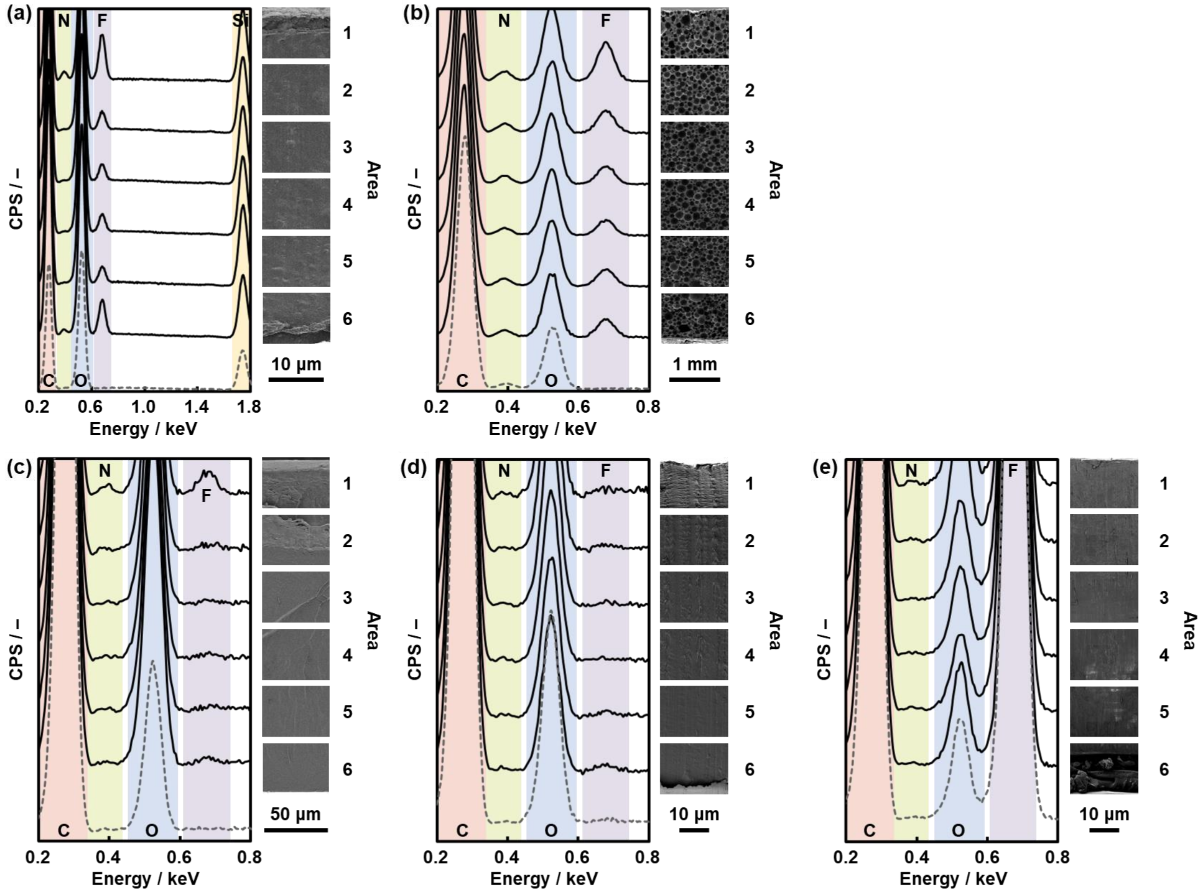

Figure S2. EDX spectra (left panel) analyzed on the selected areas 1–6 in the cross-sectional images (right panels) of SR/PPy (a), PU/PPy (b), PMMA/PPy (c), PP/PPy (d), and PTFE/PPy (e). The spectra in the bottom (dashed line) correspond to those of the bare substrate.

The quantification results of N and F in each area are summarized in Table S2. The surface and bottom layers in the areas 1 and 6 contained the larger amount of PPy compared with that in the other areas. On the other hand, for example, the areas 1 and 6 of the SR/PPy showed the peaks corresponding to Si in SR in addition to F and N corresponding to TFBQ-doped PPy (Figure S2a and Table S2). This result indicates that formation of PPy was achieved not only on the surface but also inside the substrates.

Table S2. Quantification of N and F (at%) in each area on the cross sections.

N in the areas	SR/PPy	PU/PPy	PMMA/PPy	PP/PPy	PTFE/PPy
1	2.12	4.06	1.69	0.83	0.12
2	1.06	3.10	0.37	0.53	0.09
3	0.94	3.29	0.29	0.57	0.04
4	0.95	3.17	0.23	0.59	0.00
5	0.86	3.25	0.36	0.50	0.00
6	1.99	3.45	0.49	0.09	0.00
Bare substrate	0.57 (SR)	3.29 (PU)	0.09 (PMMA)	0.24 (PP)	0.04 (PTFE)
F in the areas	SR/PPy	PU/PPy	PMMA/PPy	PP/PPy	PTFE/PPy
1	5.63	4.27	2.57	0.55	48.3
2	3.27	2.64	0.24	0.38	55.3
3	2.77	2.71	0.06	0.04	56.1
4	2.58	2.32	0.08	0.19	56.1
5	2.69	2.60	0.11	0.30	53.4
6	5.57	2.87	1.58	0.39	41.0
Bare substrate	0.02 (SR)	0.19 (PU)	0.08 (PMMA)	0.01 (PP)	57.8 (PTFE)

The area numbers correspond to those in the SEM images in Figure S2.

Quantification by CHN and halogen elemental analysis

Table S3. CHN elemental analysis.

Substrates	N / atomic %	F / atomic %	PPy / wt %
SR	0.03 ± 0.05	–	
SR/PPy	1.45 ± 0.18	4.42 ± 0.11	4.78
PU	3.14 ± 0.04	–	
PU/PPy	3.31 ± 0.15	3.60 ± 0.80	0.77
PMMA	0.07 ± 0.06	–	
PMMA/PPy	0.34 ± 0.06	1.24 ± 0.03	1.19
PP	0.00 ± 0.00	–	
PP/PPy	0.17 ± 0.04	0.08 ± 0.01	0.77

As the original substrate contained no halogen, the weight percent of C and N was measured using a conventional CHN elemental analyzer. The composite after the polymerization contained fluorine originating from the TFBQ dopant. The weight ratio of C (w_C) and N (w_N) was measured using a different system (Exeter Analysis CE-440). The weight ratio of F (w_F) was measured using a halogen analysis system (Nacsic NS-11). The weight ratio of the remaining species (O (w_O) and Si (w_{Si})) is calculated by subtracting the sum of w_H, w_C, w_N, and w_F from 1. The atomic ratio of the element i (x_i) was calculated by dividing the weight ratio (w_i) by the atomic weight. The PPy proportion was calculated by the differences between the atomic ratio of N before and after the polymerization using (Eq. S3).

UV-Vis-NIR spectra

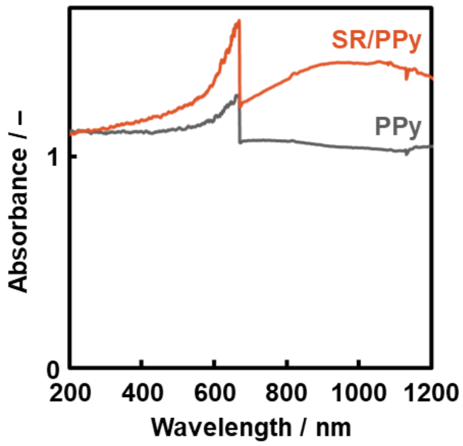

Figure S3. UV-Vis-NIR spectra of SR/PPy substrate and commercial PPy powder. The noise around 700 nm is caused by changes in the detector for visible and NIR regions.

The spectra of the resultant PPy synthesized in the free volume space of SR and PPy powder (Aldrich) were measured by the transmittance and diffuse-reflectance modes, respectively. Both the samples showed the similar absorption spectra in the entire range of UV-Vis-NIR. The results indicate that the sufficient conjugation length is achieved by the polymerization in the free volume space.

Lifetime curves of PALS and their analysis

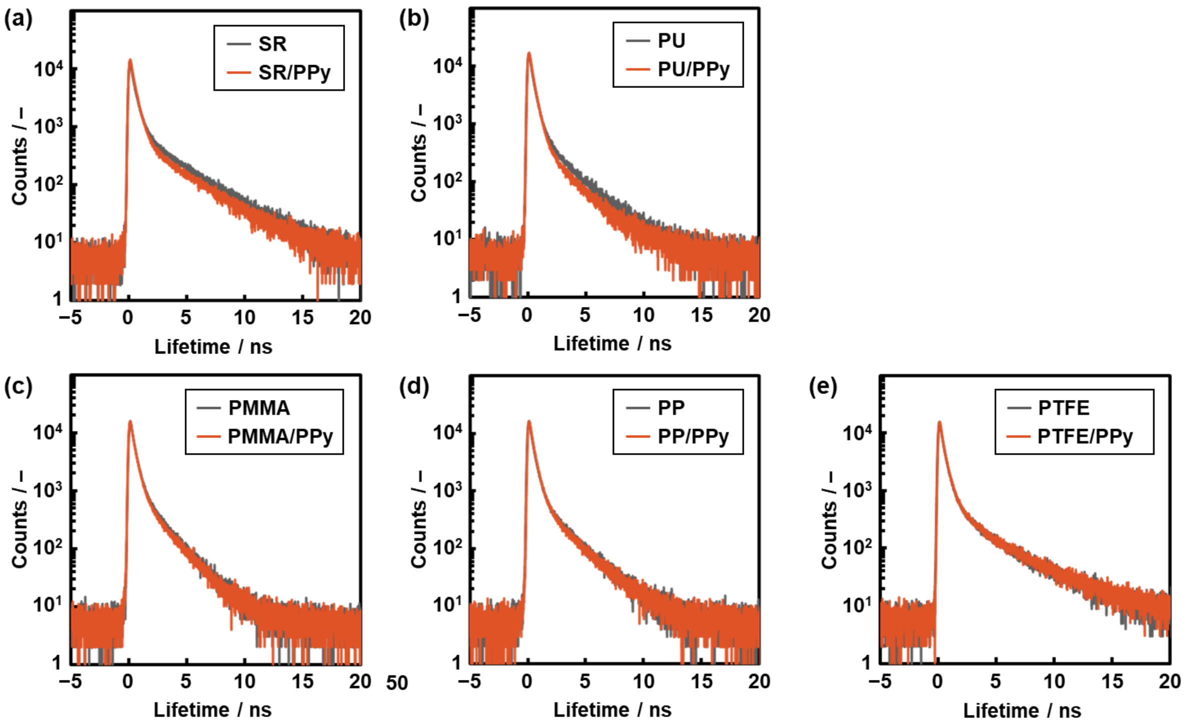

Figure S4. Lifetime curves of SR/PPy (a), PU/PPy (b), PMMA/PPy (c), PP/PPy (d), and PTFE/PPy (e).

Based on the lifetime curves, the parameters related to the free volume of polymer, such as lifetime (τ_3), pore radius (r_3), total free volume (V_f), relative intensity (I_3), and free-volume fraction (f), were calculated by the following method.

The lifetime curve was fitted using least squared method by the sum of the log-normal distribution model equation on the assumption that the life time is approximated by log-normal distribution (Eq. S4), where x is the lifetime (τ) in this case, μ and σ^2 are the mean and variance of the normal distribution, respectively.

$$f(x) = \frac{1}{\sqrt{2\pi}\sigma x} exp\left(-\frac{(lnx-\mu)^2}{2\sigma^2}\right) \quad \text{… (Eq. S4)}$$

Note that the approximation using the log-normal distribution has no chemical and physical meanings. The method is an empirically used for PALS analysis. After the fitting, the lifetime of each content, such as τ_2 (second content) and τ_3 (third content), and its standard deviation were calculated from the average and mean for each log-normal distribution, respectively. The average radius of the free volume (r_3) was calculated from τ_3 using Tao-Eldrup equation (Eqs.

S5 and S6) on the assumption of the spherical pore.[65,66]

$$\tau_3 = 0.5\left[1 - \frac{r_3}{r_0} + \frac{1}{2\pi} sin\left(\frac{2\pi r_3}{r_0}\right)\right]^{-1} \quad \text{… (Eq. S5)}$$

$$r_0 = r_3 + \Delta r \ (\Delta r = 0.166 \text{ nm}) \quad \text{… (Eq. S6)}$$

The total free volume (V_f) is calculated using (Eq. S7) on the assumption of the spherical pore.

$$V_f = \frac{4}{3}\pi r_3^3 \quad \text{… (Eq. S7)}$$

In the present work, the measurement was performed to reach the total count of the intensity up to 10^6 counts. If the total count is assumed to be 100, the relative intensity I_3 can be calculated by the integral of the intensity about τ_3. The free-volume fraction (f) was estimated from I_3 and V_3 using (Eq. S8), where A is regarded as a constant 0.0018 for polymer materials.[67,68]

$$f = V_f \times A \times I_3 \quad \text{… (Eq. S8)}$$

Penetration of Py and TFBQ

Table S4. PALS analysis of SR, SR/Py, and SR/TFBQ samples

Sample	Lifetime τ_3 / ns	Estimated pore radius r_3 / nm	Intensity I_3 / %	ΔI_3 / %
SR	3.27	0.382	31.9	–
SR/Py	3.20	0.377	30.5	–1.4
SR/TFBQ	3.30	0.383	28.4	–3.5

Only Py or TFBQ was diffused into SR in the same procedure to prepare PPy. After the SR substrate was exposed to the vapor, the sample was vacuum-dried under the same conditions. The decrease in ΔI_3 in the PALS analysis indicates that these monomeric molecules are introduced in the free volume space of SR. The results imply that the SR/PPy sample include the doped and free TFBQ. Based on Table S3, some samples had the atomic concentration of F higher than that assuming the fully doped state. In such case, free TFBQ is introduced in the matrix polymers.

Solution-phase polymerization as a reference

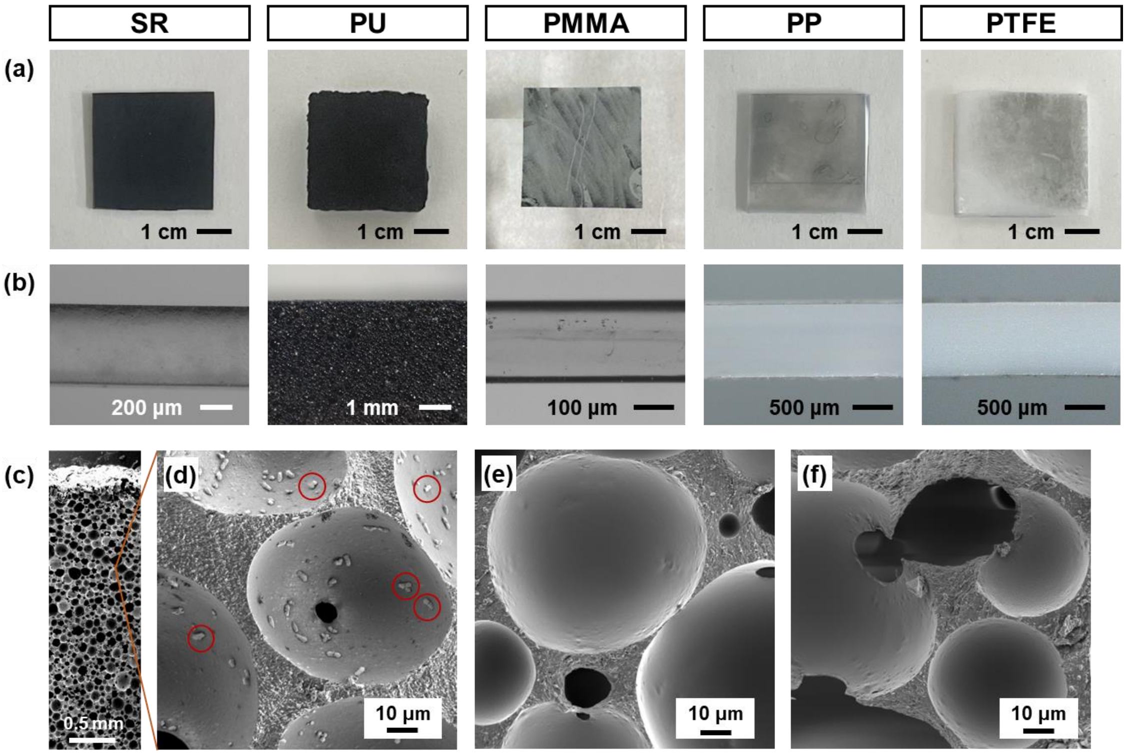

Figure S5. Solution-phase synthesis of PPy as a reference experiment in SR, PU, PMMA, PP, and PTFE. (a) Surface images. (b) Cross-sectional optical microscopy images. (c,d) Cross-sectional SEM images of PU after the solution-phase polymerization (c,d), pristine PU (e), and PU after the solution-phase polymerization (f).

The method of the solution-phase polymerization was referred to the procedure in our previous work.[25] The substrates were immersed in the neat liquid of Py for 48 h at 4 °C. The substrates were withdrawn and then immersed in the 2-propanol solution containing copper (II) chloride ($CuCl_2$) as an oxidative agent for 24 h at 25 °C. As PMMA was eluted in the monomer liquid, another method, the oligomer-mediated polymerization,[S2] was used for the synthesis. The oligomer solution was prepared by mixing Py and $CuCl_2$ in 2-PrOH and subsequent filtration to remove the precipitates. PMMA was maintained in the solution for 48 h at 25 °C. The detailed conditions were referred to the literatures.[25,S2]

The color of the surface was changed to gray and black for SR, PMMA, PP, and PTFE (Figure S5a). However, the coloration inside the substrates was not clearly observed on the cross sections compared with that for the vapor-phase synthesis (Figure S5b and Figure 2). The inside of PU shows the color change to black because the sponge structure caused the coating and deposition on the inner wall (Figure S5c,d). In particular, a large number of the PPy particles in micrometer size was formed on the inner wall (the red circles in Figure S5d). In

contrast, such deposition was not observed for the pristine PU and PU/PPy prepared by the vapor-phase polymerization (Figure S5e,f). These microscopy observation indicates that preferential formation of PPy in the free volume space was not achieved by the solution polymerization method.

Additional Reference

S2. Y. Oaki, T. Oki, H. Imai, *J. Mater. Chem.* **2018**, *22*, 21195.

Table S5. Decrease in the free volume and PPy proportions for the reference samples.

Sample	Lifetime τ_3 / ns	Estimated pore radius r_3 / nm	Intensity I_3 / %	Free volume fraction f / vol%	ΔI_3 / %	PPy / wt% (EDX)
SR	3.27	0.382	31.9	13.4	−1.0	1.37
SR/PPy	3.26	0.381	30.9	12.9		
PU	2.28	0.310	21.3	4.78	−10.5 [b]	13.8 [a]
PU/PPy	2.13	0.297	10.8	2.13		
PMMA	1.82	0.268	27.5	3.99	0.3	N. A.[c]
PMMA/PPy	1.83	0.269	27.8	4.08		
PP	2.15	0.299	23.2	4.68	0.4	0
PP/PPy	2.17	0.301	23.6	4.85		
PTFE	3.41	0.390	23.7	10.6	−0.3	0
PTFE/PPy	3.46	0.393	23.4	10.7		

[a] The accurate proportion was not calculated by the increment of nitrogen content because the original PU contained nitrogen. In addition, a large amount of the PPy particles were formed in the inner wall of the sponge structure.

[b] A significant decrease in I_3 was caused by the PPy particles more than 10 wt % deposited on the inner wall.

[c] The elemental analysis was not carried out.

Compared with the vapor-phase synthesis, the significant decrease in I_3 was not observed by the solution-phase synthesis (Table 1 and Table S5). These microscopy observations and PALS analyses indicate that the penetration of PPy in the free volume space is not achieved by the solution-phase polymerization. Therefore, the use of the monomer vapor is required to achieve the infiltration of the free-volume space.

Control of the PPy proportions in the composites

Table S6. PPy proportions in SR/PPy and PMMA/PPy with changes in the initial amount of Py and TFBQ.

Sample	Py and TFBQ / mmol	Lifetime τ_3 / ns	Estimated pore radius r_3 / nm	Intensity I_3 / %	Free-volume fraction f / vol%	ΔI_3 / %	PPy / wt% (EDX)
SR/PPy	0.1	3.24 ± 0.015	0.382 ± 0.005	28.6 ± 0.99	14.6 ± 0.9	−3.2 ± 0.99	1.78 ± 0.69
	1	3.25 ± 0.044	0.380 ± 0.003	26.7 ± 0.23	13.6 ± 0.2	−5.0 ± 0.23	2.24 ± 0.46
	10	3.29 ± 0.097	0.383 ± 0.006	24.1 ± 0.99	12.7 ± 0.6	−7.7 ± 0.99	4.11 ± 1.03
PMMA/PPy	0.05	1.89 ± 0.013	0.275 ± 0.001	23.5 ± 0.42	6.2 ± 0.1	−1.4 ± 0.42	0.60 ± 0.17
	0.1	1.85 ± 0.020	0.271 ± 0.002	24.1 ± 1.2	6.1 ± 0.2	−0.70 ± 1.2	1.10 ± 1.19
	0.5	1.84 ± 0.005	0.271 ± 0.001	22.7 ± 0.75	5.9 ± 0.1	−2.2 ± 0.75	2.50 ± 2.40
	1	1.87 ± 0.009	0.273 ± 0.001	22.5 ± 1.2	6.0 ± 0.2	−2.3 ± 1.2	3.28 ± 2.15
	10	1.85 ± 0.016	0.271 ± 0.002	22.3 ± 1.2	5.9 ± 0.1	−2.5 ± 1.2	3.92 ± 1.45

Three different samples were prepared and measured to ensure the reproducibility ($N = 3$). The PPy contents increased with increasing the initial amounts of Py monomer and TFBQ oxidant. The decrement of I_3 ($\Delta I_3 < 0$) and PPy proportion to the initial amounts of Py and TFBQ were summarized in Figure 4c.

Syntheses of the other conductive polymers

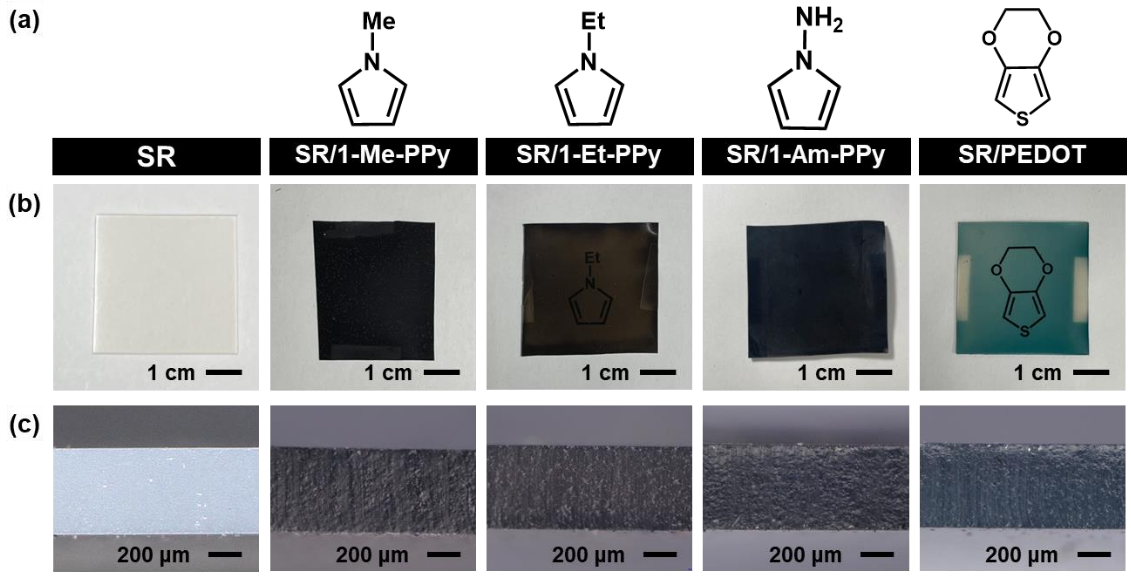

Figure S6. Polymerization of the other heteroaromatic monomers, namely 1-Me-Py, 1-Et-Py, 1-Am-Py, and EDOT, in SR. (a) Chemical structures of the monomers. (b) Surface images. (c) Cross-sectional optical microscopy images.

The coloration implies that the monomers of 1-Me-Py, 1-Et-Py, 1-Am-Py, and EDOT provided the corresponding polymers 1-Me-PPy, 1-Et-PPy, 1-Am-PPy, and PEDOT inside SR, respectively.

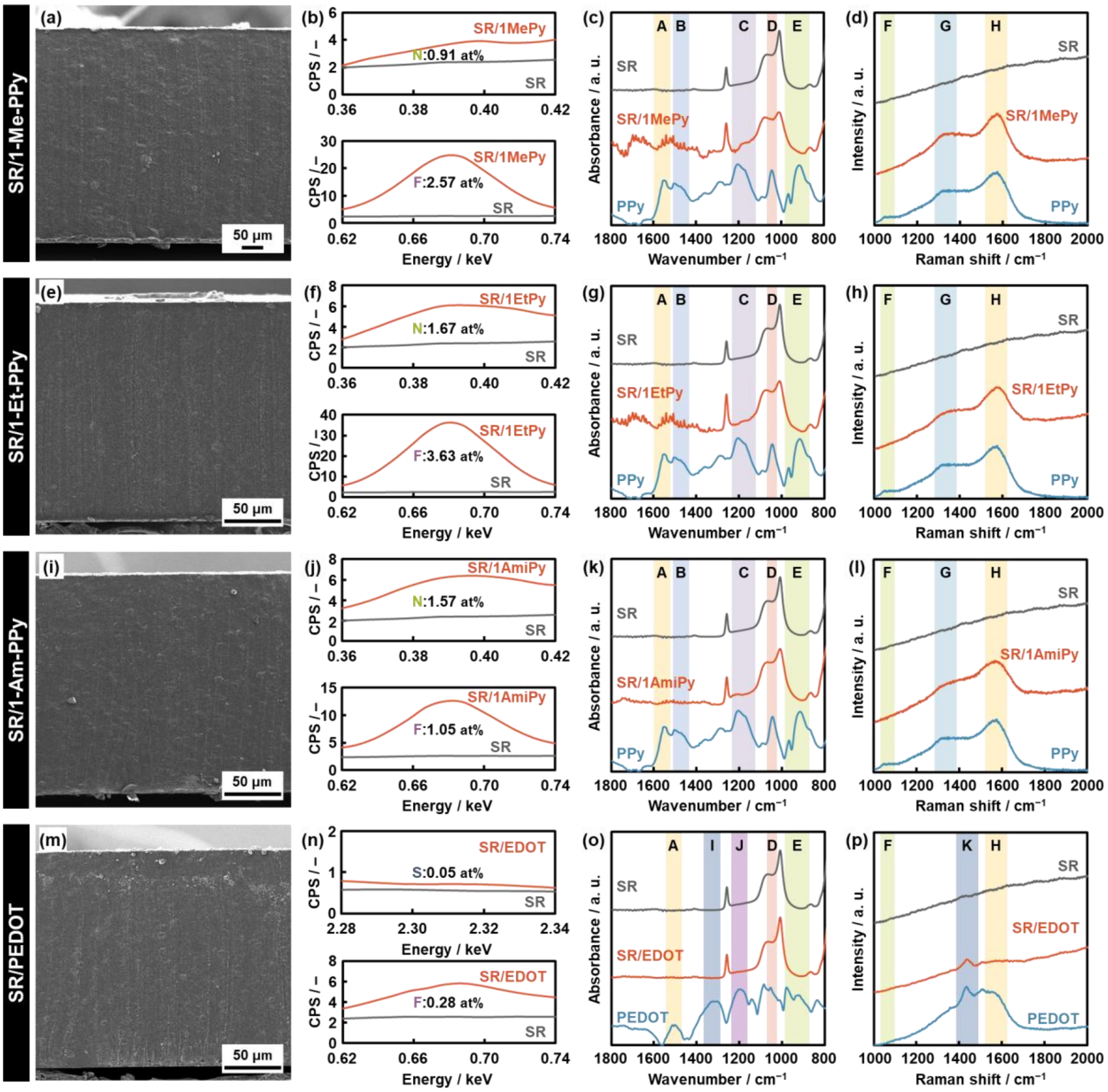

Figure S7. Structural analyses on the cross section of SR/1-Me-PPy (a–d), SR/1-Et-PPy (e–h), SR/1-Am-PPy (i–l), and SR/PEDOT (m–p). (a,e,i,m) SEM images. (b,f,j,n) EDX spectra of the bare substrates (gray) and composites (orange). (c,g,k,o) FT-IR spectra of the bare substrates (gray), composites (orange), and reference PPy (blue). (d,h,l,p) Raman spectra of the bare substrates (gray), composites (orange), and reference PPy and PEDOT (blue).

The EDX analyses on the cross sections indicate the formation of the heteroaromatic polymers with the doping of TFBQ (Figure S7a,b,e,f,i,j,m,n). Based on the quantification results, the polymer proportion was calculated according to the method in the note with Table S3 (Table S7). The photograph and EDX spectrum indicate the proportion of PEDOT in SR is lower than that of the other polymers. FT-IR and Raman spectra showed the peaks characteristic of the guest heteroaromatic polymers (Figure S7c,d,g,h,k,l,o,p). The assignment of the peaks A–E is

described in the main text. In addition, the peaks I and J for PEDOT were assigned to the C–O and C–S stretching vibrations, respectively. The Raman peaks F–H correspond to the C–H, C–N, and C=C bonds characteristic of PPy, respectively. The additional peak K for PEDOT was assigned to C–S bond. When the proportion of PPy was not so large, the signals characteristic of PPy, such as N for EDX and absorption for FT-IR, were weak. On the other hand, Raman spectra showed the presence of PPy in the observed area clearly.

Table S7. Decrease in the free volume with incorporation of the guest polymers in SR.

Sample	Lifetime τ_3 / ns	Estimated pore radius r_3 / nm	Intensity I_3 / %	Free-volume fraction f / vol%	ΔI_3 / %	Polymer / wt% (EDX)
SR	3.27	0.382	31.9	15.9	–	–
SR/1-Me-PPy	3.19	0.376	28.6	14.0	−3.3	1.44
SR/1-Et-PPy	3.19	0.376	27.5	13.5	−4.4	4.18
SR/1-Am-PPy	3.20	0.377	26.6	13.3	−5.3	3.84
SR/PEDOT	3.22	0.378	27.2	13.6	−4.7	0.38

The PALS data was analyzed by the same method described with Figure S3. The decrease in I_3 indicates the decrease in the free volume space with infiltration of the guest polymer (Table S7). The polymer proportion was calculated based on the EDX quantification results of N for 1-Me-PPy, 1-Et-PPy, and 1-Am-PPy and S for PEDOT. Based on these results (Figures S4, S5, and Table S7), the vapor-phase synthesis of 1-Me-PPy, 1-Et-PPy, 1-Am-PPy, and PEDOT was achieved in the free volume space of SR.

Reproducibility of the mechanical properties

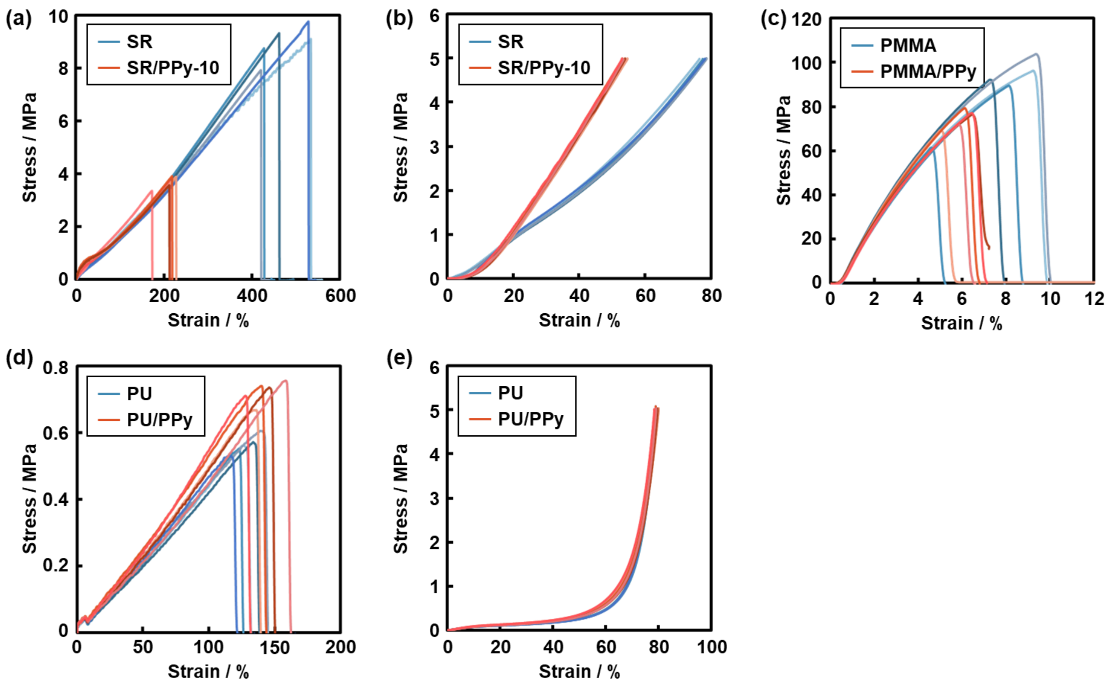

Figure S8. Mechanical properties of SR/PPy (a,b), PMMA/PPy (c), and PU/PPy (d,e). (a,c,d) Stress-strain curve of the tensile tests ($N = 5$). (b,e) Stress-strain curve of the compression tests ($N = 5$).

Based on these results, the representative data were displayed in Figure 5. The average values were calculated using these data in Figure S8.

Permeation rate of water vapor

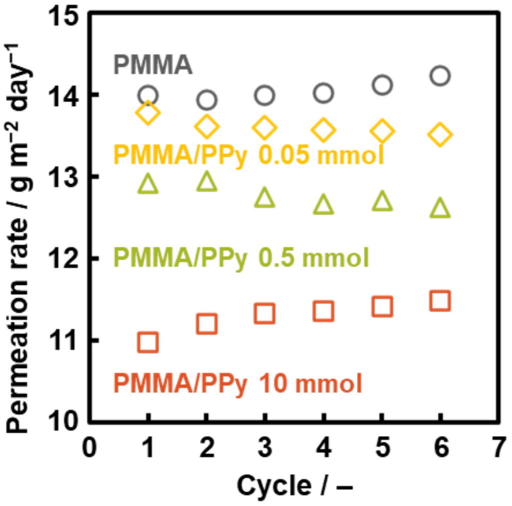

Figure S9. Relationship between the cycle and permeation rate of water vapor for PMMA/PPy prepared with the different initial amount of Py and TFBQ (0.05, 0.5, and 10 mmol).

The average values were calculated for each sample using the rates in six cycles.

Mechanical properties of SR/PPy

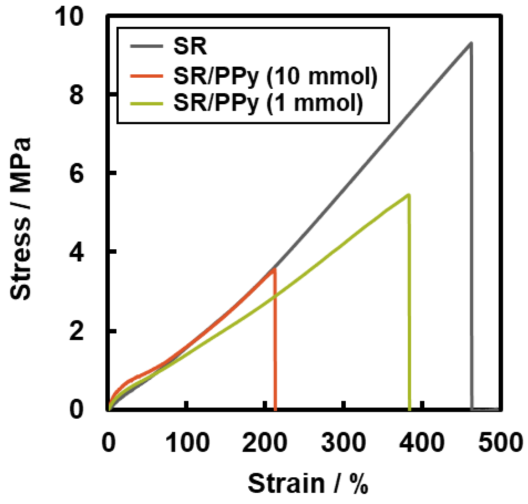

Figure S10. Stress-strain curves of SR/PPy prepared with changes in the initial amount of PY and TFBQ. The amount in the original condition (10 mmol) was changed to 1 mmol for preparation of the conductive rubber.

The tensile strength was 8.97 ± 1.2 MPa for the original SR, 3.69 ± 0.40 MPa for SR/PPy (10 mmol), and 5.78 ± 1.6 MPa for SR/PPy (1 mmol) (N = 5). The break elongation was 476 ± 97 % for the original SR, 209 ± 36 % for SR/PPy (10 mmol), and 388 ± 118 % for SR/PPy (1 mmol). The results indicate that the mechanical properties are changed by the amount of the supplied monomer and oxidant. The rubbery nature of SR is preserved with the decrease in the amounts of the supplied monomer and oxidant.

Responsivity of SR/PPy and PU/PPy

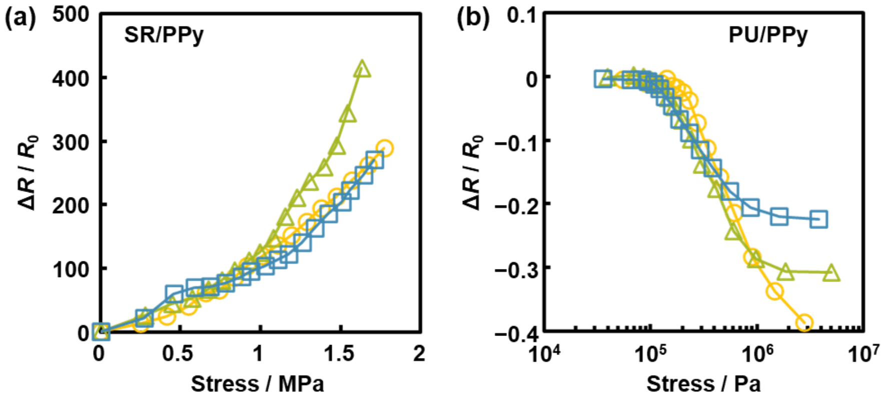

Figure S11. Responsivity of the SR/PPy and PU/PPy conductive rubbers to the tensile and compression stresses, respectively. (a) Relationship between the tensile stress and ΔR / R_0 of the three SR/PPy samples. (b) Relationship between the compression stress and ΔR / R_0 of the three PU/PPy samples.

As the responsivity (ΔR / R_0) to the applied stresses was reproducible ($N = 3$), the average and standard deviation were plotted in Figure 6a,b.

Stress-strain curves of the softness models

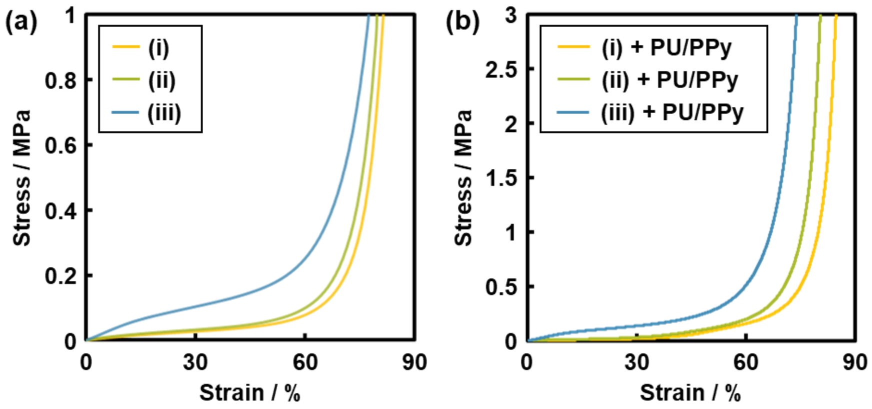

Figure S12. Stress-strain curves of the compression tests using softness models (i)–(iii) (a) and PU/PPy conductive rubber between the softness models (i)–(iii) (b).

The stress-strain curve in Figure S12b correspond to that for the setup in Figure 6e. The measurement of the softness was carried out at strain 70 % (Figure S12b).

PU/PPy sample for sensing compression stresses

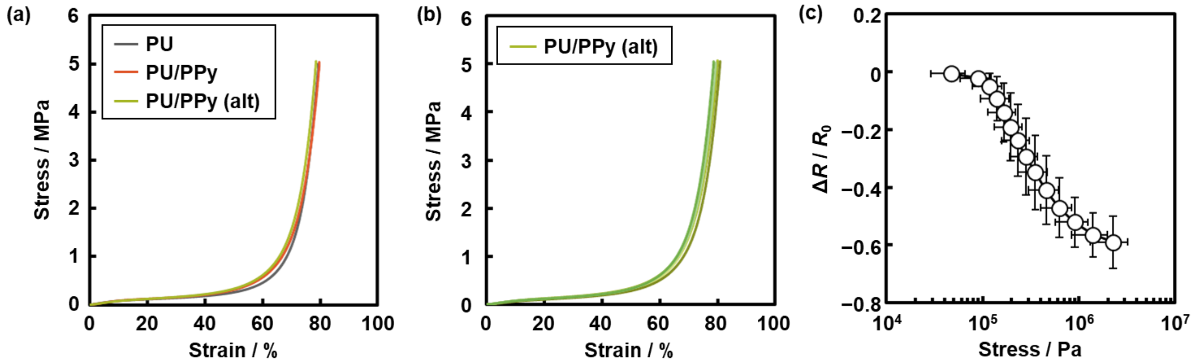

Figure S13. Mechanical properties and responsivity of the PU/PPy samples prepared by the alternative supply of Py and TFBQ vapor (PU/PPy (alt)). (a) Stress-strain curves of PU, PU/PPy, and PU/PPy (alt) of the compression test. (b) Stress-strain curves of PU/PPy (alt) samples of the compression test to ensure the reproducibility (N = 3). (c) Relationship between the compression stress and ΔR / R_0 of the PU/PPy (alt) samples (N = 3).

PU/PPy was prepared by the different method to improve the conductivity and responsivity. The Py and TFBQ vapor was alternatively exposed to PU (See the method section). The stress-strain curve was not changed by changes of the preparation method (Figure S13a). The mechanical properties were reproducible for three samples (N = 3) (Figure S13b). The responsivity was improved compared with that in Figure 6b (Figure S13c). The more homogenous infiltration of PPy is achieved by the alternative exposure method. The PU/PPy (alt) sample was used to measure the differences in the softness of the model tracts (Figure 6e,f).
